# 

**Published:** 1903-10-03

**Authors:** 


### CONTENTS.

**The Cost of Small-pox** - 1

**The Public Health and the Duty of the Daily Press** - 2

**Annotations** - 3

**Notes and News** - 4

**The Progress of Cancer Research** - 5

**Hospital Clinics and Medical Progress—**

The Prevention of Heart Disease in Children - 10

The Urban Treatment of Surgical Tuberculosis - 12

When to Operate in Cleft Palate - 12

Post typhoid Sepsis - 13

The Surgical Treatment of Colitis - 13

Cerebro-spinal Fluid in General Paralysis - 14

Progress in Dermatology - 14

Progress in Surgery of the Pancreas and Spleen - 15

Progress in Surgery of the Stomach - 16

Progress in Phototherapy and Electro-therapeutics - 16

**New Appliances and Things Medical** - 19

**Medical Teaching in the Fifteenth Century** - 20

**Hospital Administration—**

The Sunderland Union Infirmary - 21

Modern Hospital and Institutional Fittings - 22

Visits to Private Asylums - 23

Glances at the Hospitals - 23

*NURSING SECTION.*

**Notes on News from the Nursing World—** Our Christmas Distribution—The Half-Trained Nurse in Paris—A New Departure at Guy's Hospital—King's College Hospital—Royal Free Hospital—Royal Hospital for Women and Children—The North-Western Poor Law Conference and Nursing—Central Midwives Board—Conference of American Superintendents of Training Schools—Nurses and Public Worship—York Nurses in Boarded off Cribs—To Help Nurses in their Work—Garden Party at Glasgow—The Question of a Specialist's Fee—Night Duty at Newton Abbott—Short Items - 1-4

**Lectures on Ophthalmic Nursing** - 5

**Nursing by Sisters of Mercy** - 6

**Hints on How to Start a District Nursing Association** - 8

**Sketches at a Children's Hospital** - 9

**Nursing under the Municipality in Paris** - 11

**Beyond the Seas: Nursing in a Cawnpore Hospital** 12

**Charing Cross Hospital** - 15

**The Prevention of Bed Sores** - 16

**To Nurses** - 16

**Echoes from the Outside World** - 17

**A Book and its Story** - 18

**Everybody's Opinion** - 19

**Appointments** - 19

**Novelties for Nurses** - 20

**Presentations** - 21

**"The Hospital" Convalescent Fund** - 21

**Where to Go** - 21

**Travel Notes and Queries** - 21

**Notes and Queries** - 21

**For Reading to the Sick** - 22

